# Application of an updated severity‐based FMEA approach to Radiation Therapy QA: A multi‐institutional exercise

**DOI:** 10.1002/acm2.70674

**Published:** 2026-08-13

**Authors:** Alejandra Rangel, Ryan Studinski, Ady Abdellatif, Leigh Conroy, Jenna King, Humza Nusrat, Michael Oliver

**Affiliations:** ^1^ Medical Physics Department Trillium Health Partners ‐ Credit Valley Hospital Mississauga Ontario Canada; ^2^ Department of Physics Juravinski Hospital and Cancer Centre at Hamilton Health Sciences Hamilton Ontario Canada; ^3^ Medical Physics Department R. S. McLaughlin Durham Regional Cancer Centre Lakeridge Health Oshawa Ontario Canada; ^4^ Radiation Medicine Program Princess Margaret Cancer Centre Toronto Ontario Canada; ^5^ Medical Physics Department, Hudson Regional Cancer Program (North Simcoe Muskoka) Royal Victoria Regional Health Centre Barrie Ontario Canada; ^6^ Department of Radiation Oncology Henry Ford Health Detroit Michigan USA; ^7^ Medical Physics Department, Shirley and Jim Fielding Northeast Cancer Center Health Sciences North Sudbury Ontario Canada

**Keywords:** action priority, FMEA, multi‑institutional study, quality management, risk priority number

## Abstract

**Background:**

External beam radiation therapy (RT) involves many complex steps to safely and reliably treat patients. The radiation oncology community has adopted process maps and failure modes and effects analysis (FMEA) to analyze risk using the TG‑100 methodology, but the traditional TG‑100–endorsed risk priority number (RPN) has recognized limitations. An updated risk‑profile introduced by the automotive industry, action priority (AP), offers a severity‑weighted alternative that may better support risk‑based decision‑making in RT.

**Purpose:**

To compare the traditional RPN‐based FMEA approach with the updated AP‐based approach for identifying high‐risk potential failure modes (PFMs) in a representative external beam RT workflow across multiple institutions, and to assess whether the AP framework provides additional value to the radiation oncology community.

**Methods:**

Nine physicists from different cancer centers in Ontario, Canada collaboratively developed and refined a list of 63 PFMs associated with a generic RT workflow requiring data transfer. Each PFM was scored for occurrence (O), severity (S), and detectability (D) using TG‑100 recommendations. Three scoring scenarios were evaluated: average, worst‑case, and a severity‑focused score. High‑risk PFMs were identified using the traditional (top RPN quartile and/or S ≥ 8) and the updated (High or Medium AP categories) FMEA criteria.

**Results:**

Seven high‑risk PFMs were selected using the updated FMEA, and an additional 23 were selected using the traditional FMEA. All PFMs identified by the updated FMEA approach were also selected by the traditional FMEA approach; however, the traditional FMEA approach identified many additional PFMs due to its quartile‑based and severity‐threshold criteria. The updated AP approach appeared less affected by the scoring variability which may reduce the need for consensus discussion, it was generally easier to apply, showed more consistent behaviour across FMEA iterations and provided a clearer action guidance.

**Conclusions:**

The use of the updated AP approach in this study suggests a clearer, severity‐driven, and more streamlined strategy to prioritize high‐risk PFMs, potentially reducing the burden of multi‑institutional FMEA while maintaining consistent identification of critical risks.

## 1 INTRODUCTION

Improving the quality and safety of radiation treatment delivery is one of the major roles of medical physicists in radiation therapy (RT).^1^ As clinical workflows have become increasingly complex, structured methods for proactively identifying and mitigating risk have gained importance. Failure modes and effects analysis (FMEA) is a risk analysis tool originally developed for military and industrial applications that has been widely adopted across healthcare for this purpose. The value of FMEA in improving safety culture, identifying vulnerabilities, and strengthening quality management practices has been demonstrated in numerous studies and clinical settings.^2^, ^3^, ^4^, ^5^

In 2016, TG‐100 introduced an FMEA methodology tailored specifically to the field of Medical Physics^6^, incorporating process mapping, failure mode identification, and quantitative scoring of occurrence (O), severity (S), and detectability (D). The TG‐100–endorsed risk priority number (RPN), calculated as the product O × S × D, has since become the dominant method for prioritizing potential failure modes (PFMs) in RT.^7^, ^8^, ^9^, ^10^, ^11^ However, the widespread use of the RPN in fields that adopted it earlier than Medical Physics has revealed several important limitations.^12^, ^13^, ^14^

**FIGURE 1:**
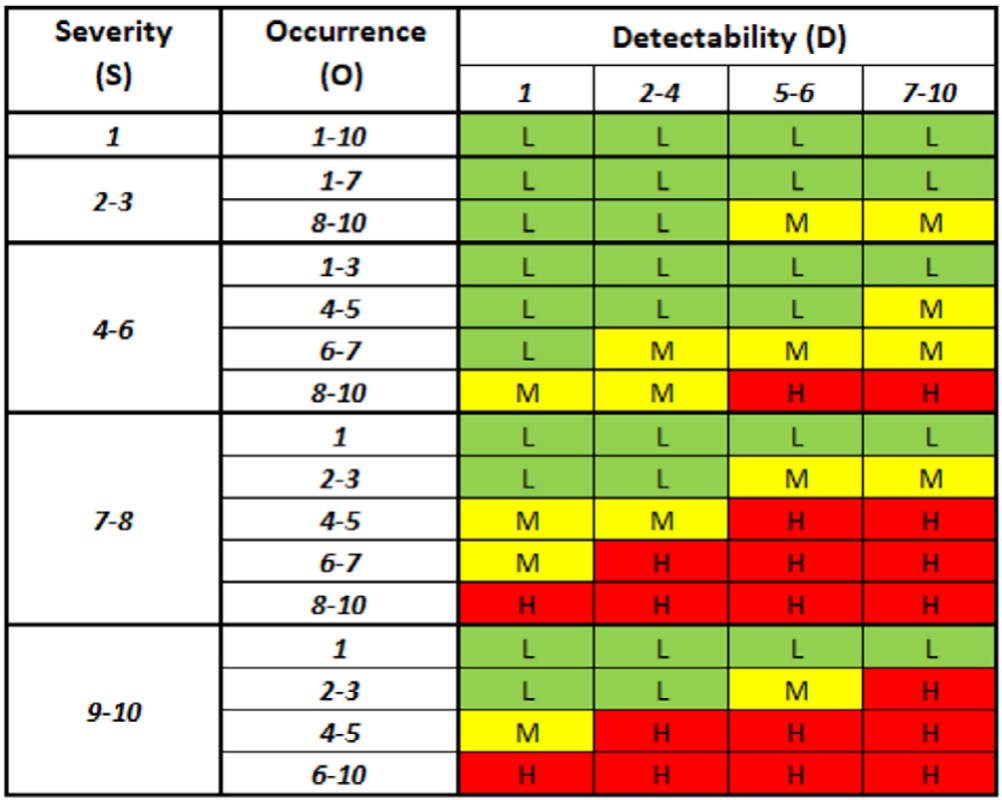
Summary of action priority categories; adapted from definitions in AIAG & VDA FMEA Handbook.^17^

In response to these concerns, the Automotive Industry Action Group (AIAG),^15^ and the German Association of the Automotive Industry (Verband der Automobilindustrie, VDA)^16^ introduced an updated risk‑profiling framework in 2019 known as action priority (AP).^17^ Using the AP, failure modes are classified into low, medium, or high AP categories via a look‐up table that weights severity most heavily and detectability least (Figure 1). PFMs categorized as high AP are those with the highest priority for review, and action must be taken to reduce risk. Medium AP PFMs are of the second highest priority for review and action should be taken to reduce risk while low AP PFMs require only on‐going monitoring. This alternative approach is quickly gaining acceptance beyond the automotive industry^18^, ^19^, ^20^, ^21^ and only recently has begun to appear in RT literature.^22^ Thus, the potential advantages of the AP approach in RT remain largely unexplored.

To date, no study has directly compared the RPN with the AP framework when applied to the same RT process map and failure mode list. As a result, it remains unclear whether the AP:

- Identifies a similar set of high‑risk PFMs,
- Reduces the burden of consensus‑driven scoring,
- Improves consistency across multiple institutions, or
- Provides clearer guidance for action in RT.

Addressing this gap is important because the effectiveness and reproducibility of the FMEA depend heavily on the clarity and efficiency of the underlying risk‑ranking methodology.

A group of medical physicists from several cancer centers in Ontario was tasked through a volunteer‐based provincial community of practice to perform an FMEA of a representative RT external beam delivery process with the objective of evaluating the role and value of patient‐specific quality assurance (PSQA). In accordance with standard FMEA methodology and the aims of the project, the task group compiled a list of PFMs that could affect treatment accuracy and might be detectable through PSQA.

As an initial step, the group performed an FMEA of the RT delivery workflow in the absence of PSQA, establishing a baseline against which the potential value of PSQA could be evaluated. Although the analysis was originally intended to follow the traditional TG‑100‑endorsed FMEA approach only, the group became aware of the updated FMEA used widely in the automotive industry. This prompted an expanded objective: to evaluate whether the AP framework offers practical advantages—in clarity, efficiency, or consistency—over the RPN when applied to a multi‑institutional FMEA.

The present work summarizes the findings of this comparative evaluation and describes the methodological challenges and implications for future FMEA practice in radiation oncology.

## 2 METHODS

### 2.1 Design of the process map and PFMs

A multi‐institutional working group consisting of nine physicists from different cancer centers in the province of Ontario, Canada developed a generic RT process map of a representative treatment facility. The scope included photon IMRT/VMAT (including SBRT) treatments delivered with a separate, non‐integrated record‐and‐verify system, which requires transfer of data from the treatment planning system (TPS) to the oncology information system (OIS), and excluded specialized techniques and equipment such as brachytherapy, orthovoltage, MR‐Linac, stereotactic radiosurgery, Gamma Knife, Cyber Knife, and Tomotherapy.

An initial broad list of PFMs was compiled by the working group based on professional experience and then reviewed and refined to ensure that the PFMs included for scoring were appropriate, descriptive, and unambiguous by group consensus. PFMs that would have no reasonable chance of ever being identified by PSQA were excluded from the list as beyond the intended scope of this investigation. PFMs that included treatment machine performance variations remaining within typical tolerance levels as per standard accepted quality assurance (QA) guidelines were also omitted. Once this preliminary list was determined, it was then supplemented and expanded by surveying relevant literature and comparable works.^23^, ^24^, ^25^ The working group convened approximately once per month, with the development of the FMEA, scoring, and data analysis occurring over a roughly three‐year period from fall 2022 to fall 2025.

### 2.2 Scoring of PFMs

Scoring of the PFMs was done using the scoring table from TG‐100^6^ for occurrence (O), severity (S) and detectability (D). For this work, D was defined to be the detectability by a QA program fully compliant with Canadian Partnership for Quality Radiotherapy (CPQR), a thorough Physics chart check with a point dose calculation, and therapist pretreatment checks but excluding any further PSQA.

Each physicist first scored the PFMs individually and then the results were compiled and discussed as a group to ensure consistent interpretation, particularly when the standard deviation in any of the three scores was greater than three. During group discussion, evaluators clarified their interpretation of the failure mode, described local workflow differences contributing to their scoring, and reviewed relevant sections of the TG‑100 scoring rubric to promote consistency.

Following discussion, each physicist was given the opportunity to revise their scores; however, no evaluator was required to change their rating. Final scores were not forced to consensus, and the recorded values for each failure mode remained the original or revised individual scores contributed by each evaluator. This approach preserved inter‑institutional differences while ensuring that all high‑variability items had been reviewed for interpretive consistency.

Multiple scoring scenarios were used to account for institutional variability in failure mode interpretation and scoring. The scenarios were defined as follows:

- *Average* score: Calculated using the mean score from all evaluators for each potential failure mode for O, S, and D.
- *Worst‐case* score: Calculated using the highest score given by any single evaluator for each potential failure mode for O, S, and D.
- *Severity‐focused* score: Calculated using the minimum O score, maximum S score, and average D score across all evaluators.

Evaluating average, worst‑case, and severity‑focused scores^26^ allowed the analysis to remain robust to evaluator differences and ensured that high‑risk failure modes were not overlooked due to averaging effects or institutional biases.

Once scores were calculated under the three case scenarios described above, the results were analyzed via the two distinct risk‑profile frameworks of interest. The first followed the methodology outlined in TG‑100^6^ and employed the RPN, herein referred to as the *Traditional FMEA* approach, and the second employed the AP risk profile as described by the AIAG and VDA update^17^, and is herein referred to as the *Updated FMEA* approach.

### 2.3 Traditional FMEA Approach: RPN

In the traditional FMEA approach, an RPN is calculated by multiplying O, S, and D final scores. Given the range of the individual factors (from 1 to 10), the RPN of each PFM can range from 1 to 1000. RPNs are then ranked from highest to lowest in order to identify the highest risk PFMs in the process. As suggested in TG‐100, PFMs that were in the top quartile of RPN scores and/or with a severity score greater than or equal to eight were deemed to be high risk profile PFMs.

### 2.4 Updated FMEA Approach: AP

In the updated FMEA approach, the same PFMs were analyzed based on an AP risk profile.^17^ The AP of each PFM was assigned by a look‐up table (Figure 1) and each PFM was categorized into one of three levels of priority: High, Medium, or Low. Each of these levels provides a clear prioritization of the need for action by specifying when actions *must*, *should*, or *could* be implemented to reduce risk. As suggested by the AIAG and VDA update,^17^ PFMs with an AP score in the High or Medium category using this approach were deemed High Risk Profile PFMs.

## 3 RESULTS

**FIGURE 2:**
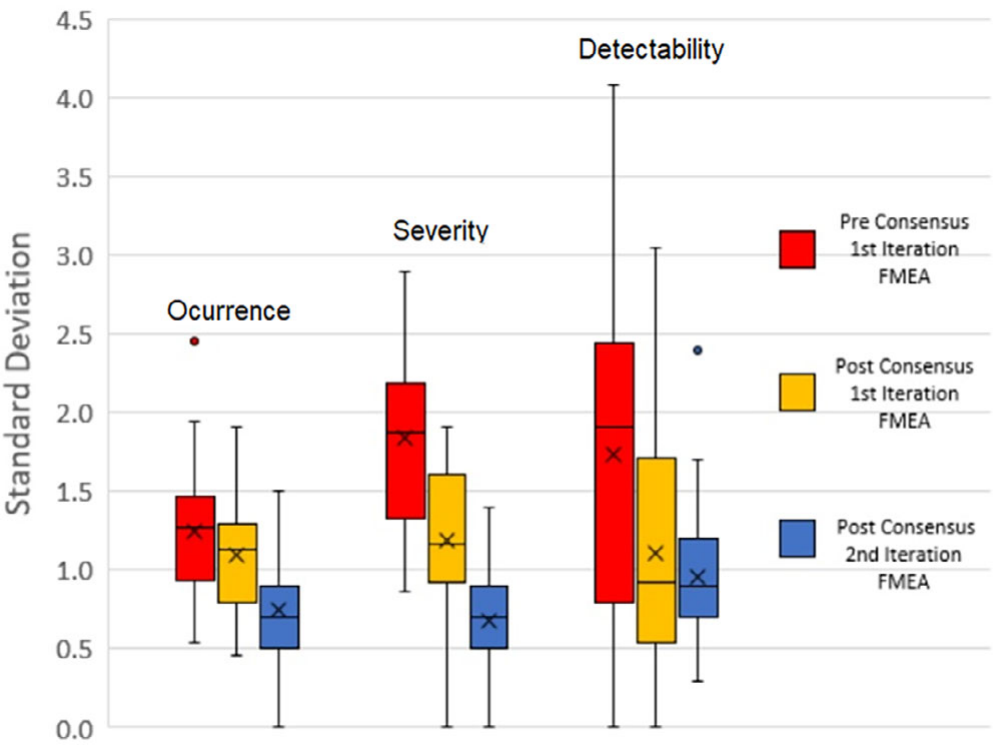
Box and whisker plot showing the distribution of the standard deviations of occurrence, severity and detectability scores over repeated iterations of the working group's FMEA process. The x inside each box represents the mean and the line represents the median.

A list of PFMs was generated from the generic process map. As intended, PFMs that would have no reasonable chance of ever being identified by PSQA were intentionally excluded, resulting in a final list of 63 PFMs (Appendix A). Although the group reached agreement on the interpretation of each PFM, consensus was not achieved for all scoring elements. Coming from different institutional perspectives, consensus was easier to achieve on severity and occurrence than on detectability, even after group discussion. The variability in scoring across iterations is illustrated in Figure 2, which shows the distribution of standard deviation values (from the list of PFMs) for each factor O, S, and D.

The first iteration used the original FMEA score from the working group, first with members working in isolation, then again after a consensus meeting. Some PFMs were deemed redundant during this meeting, thus the original list of 46 was reduced to 37 PFMs within the first iteration. The second iteration was done after the working group reviewed the literature and modified the PFMs to address concerns that had been missed; this is the iteration described in this publication.

**FIGURE 3:**
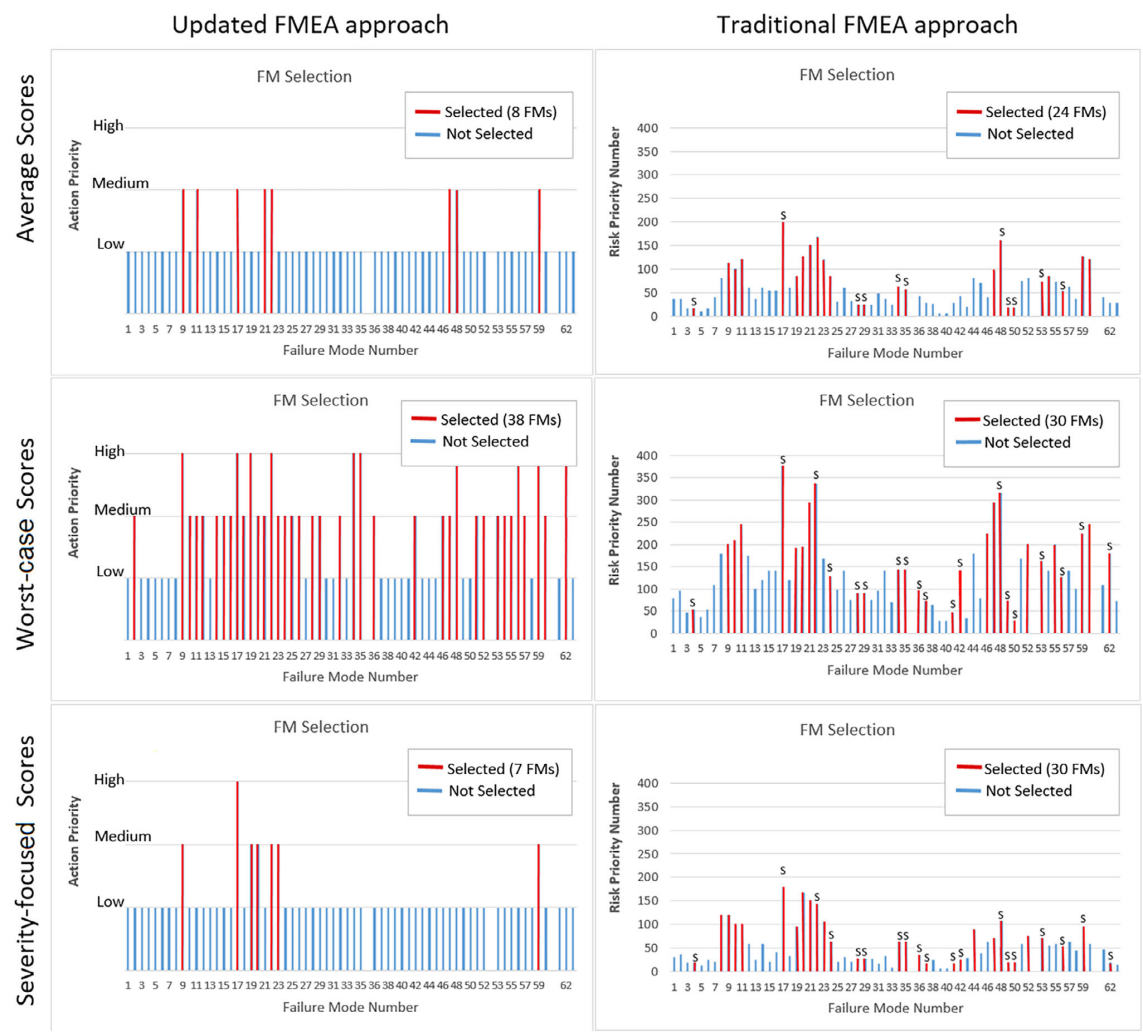
Failure mode selection when using the AP (column on the left) or the RPN top quartile + severity≥8 (column on the right) for each of the 63 PFMs under different case scenarios (see each row). For the traditional approach, those PFMs with S ≥ 8 have an “S” on top of the bar display.

Using the average scores, the traditional FMEA approach resulted in RPNs ranging from 6 to 200. Following convention, a total of 24 PFMs were selected: 15 PFMs were in the top quartile of the RPN distribution and 9 additional PFMs (not in the top quartile) had S ≥ 8. The updated FMEA approach resulted in a list of 8 PFMs with AP = Medium, 55 PFMs with AP = Low and no PFMs with AP = High. Also following convention, all 8 PFMs with AP = Medium were selected. See average‐score row in Figure 3.

Using the worst‐case scores, the RPN distribution ranged from 27 to 378. The list of selected PFMs increased to 30, 2 PFMs previously chosen by the average‐case scenario's top quartile were substituted for different PFMs and 15 additional PFMs (not in the top quartile) had S ≥ 8. Using the worst‐case scenario, the updated FMEA approach consistently selected all 8 PFMs from the average scenario plus an additional 30 PFMs (combination of Medium + High), see worst‐case scores row in Figure 3.

Using the severity‐focused scores, the distribution of RPNs ranged from 7 to 180. A total of 30 PFMs were selected: 16 from the top quartile and an additional 14 with severity scores ≥8. Unlike the other two case scenarios, the severity‑focused scenario produced 16 PFMs in the top RPN quartile rather than 15. This occurred because several PFMs shared identical RPN values despite being derived from different combinations of severity, occurrence, and detectability factors. Because the top quartile is defined by dividing the dataset into four equal portions, a total of 63 PFMs yields a threshold that can include either 15 or 16 PFMs, depending on how tied RPN values cluster at the upper end of the distribution. Using the severity‐focused scenario, the updated FMEA approach selected 6 PFMs with AP = Medium and 1 PFM with AP = High, see severity‐focused scores row in Figure 3.

**FIGURE 4:**
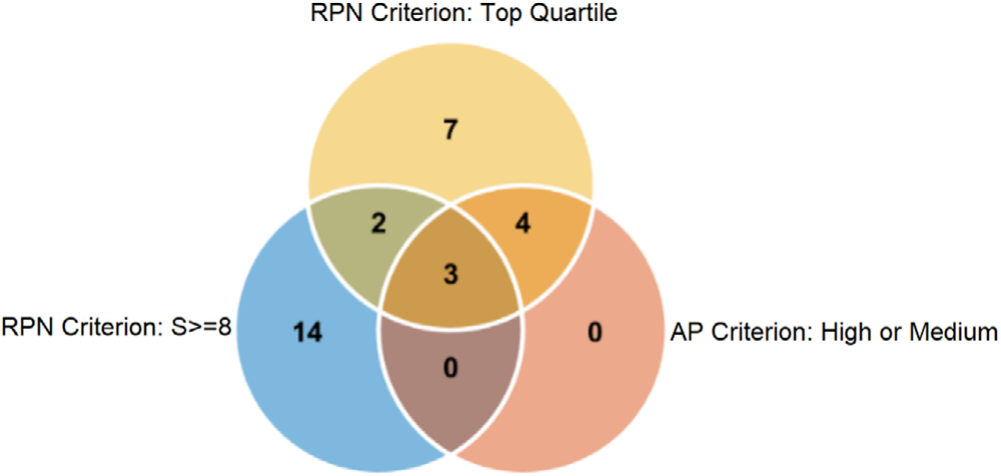
Venn diagram demonstrating the selection of high‐risk PFMs using traditional FMEA approach and the updated FMEA approach.

The traditional FMEA approach and the updated FMEA approach profiles agreed best in the selection of high priority PFMs using the average and the severity‐focused scenarios while the severity‐focused scenario also stratified PFMs into a high action level. For this reason, the data analysis was undertaken using a severity‐focused scenario, see Figures 3 and 4.

Table 1 presents the list of PFMs selected by the traditional and updated FMEA approaches using the severity‐focused scenario. NS = Not Selected by the criterion.

**TABLE 1:** PFMs selected by each analysis approach.

High risk potential failure modes		Category	Frequency	Updated	Traditional	
				AP	RPN	S ≥ 8
1	Treatment plan errors: Target contours have stray voxels.	*Planning*	*Patient specific*	*High*	*180*	*9*
2	Treatment plan too complex: Small leaf gap.			*Medium*	*168*	*NS*
3	Treatment plan not calculated on the correct version of CT scan: Older scan after weight loss.			*Medium*	*144*	*8*
4	Wrong template used to create plan: Beam parameters (e.g., incorrect energy, FFF vs FF, etc.).			*Medium*	*120*	*NS*
5	Treatment plan not calculated on correct version of CT scan: Wrong breathing phase			*Medium*	*105*	*NS*
6	Treatment plan too complex: High MU factor (MU/Gy above typical).			*Medium*	*96*	*NS*
7	Plan modified after treatment has begun (including first‐fraction treatment checks): Moderate dosimetry change.	*Documentation*	*Patient specific*	*Medium*	*96*	*8*
8	Treatment plan too complex: Fields (defined by jaws) smaller than modeled.	*Planning*	*Patient specific*	*NS*	*150*	*NS*
9	Wrong template used to create plan: Linac characteristics (e.g., SBRT vs. standard Linac).			*NS*	*120*	*NS*
10	Collision with a patient.	*Delivery*	*Patient specific*	*NS*	*108*	9
11	Wrong template used to create plan: Target optimization parameters.	*Planning*	*Patient specific*	*NS*	*100*	*NS*
12	Wrong template used to create plan: OAR optimization parameters.			*NS*	*100*	*NS*
13	Linac can't deliver plan or interlocks (e.g., machine related, MLC speed or dose rate, unable to clear).	*Linac*	*Patient specific*	*NS*	*90*	*NS*
14	Linac performance is not the same as commissioning: Other VMAT parameters like gantry speed or dose rate.	*Linac*	*Systemic*	*NS*	*75*	*NS*
15	Wrong plan version chosen for treatment before pretreatment PSQA: Major dosimetry change.	*Documentation*	*Patient specific*	*NS*	*72*	*9*
16	Collision with Linac components (e.g., OBI arms colliding with couch).	*Delivery*	*Patient specific*	*NS*	*72*	*NS*
17	Errors in commissioning/beam model: Bank A is bank B in TPS and reversed in OIS.	*TPS*	*Systemic*	*NS*	*NS*	*9*
18	Configuration difference between plan and Linac (e.g., gantry or collimator angle).	*Linac*	*Systemic*	*NS*	*NS*	*9*
19	Configuration difference between plan and treatment couch (e.g., tabletop movement, couch kick).			*NS*	*NS*	*9*
20	Gross failure of TPS: Failing to recalculate MU after parameter change (e.g., 60 Gy/30 changed to 60 Gy/8 but no MU update).	*TPS*	*Patient specific*	*NS*	*NS*	*9*
21	Gross failure of TPS: Failing to update dose distribution after parameter change (e.g., energy changed from 6 to 18MV).			*NS*	*NS*	9
22	Major Linac failure (e.g., wrong flattening filter moves into place).	*Linac*	*Systemic*	*NS*	*NS*	*9*
23	Wrong physical component installed on Linac (x‐low and x‐high flattening filters reversed or Linac Pb shielding left out).			*NS*	*NS*	*9*
24	Wrong plan version chosen for treatment after pretreatment PSQA: Major dosimetry change.	*Documentation*	*Patient specific*	*NS*	*NS*	*9*
25	Major data transfer error while importing into OIS (e.g., beam not transferred to the OIS).	*Data Transfer*	*Patient specific*	*NS*	*NS*	*9*
26	Treatment plan not calculated on correct version of CT scan: Wrong bladder filling.	*TPS*	*Patient specific*	*NS*	*NS*	*8*
27	Major degradation of linear accelerator: ion chamber leaking.	*Linac*	*Systemic*	*NS*	*NS*	*8*
28	Major degradation of linear accelerator: target melting.			*NS*	*NS*	*8*
29	Linac performance above action level: MLC calibration offset of ∼3 mm.			*NS*	*NS*	*8*
30	Linac performance above action level: MLC calibration gain of ∼1 mm/5 cm.			*NS*	*NS*	*8*

## 4 DISCUSSION

The present multi‐institutional analysis compared the traditional RPN approach with the updated AP framework for identifying high‐risk PFMs in a representative external beam RT process. Our results indicate that, within the context of this representative workflow, the two frameworks identify the same highest‐ranked PFMs (see rows 1–7 in Table 1) with additional PFMs identified by the traditional FMEA approach. It is important to note however, that this observed agreement is not entirely independent. Both frameworks are based on the same O, S, and D scores and both favor the selection of high severity PFMs. As such, a degree of overlap may be expected due to the structural alignment. Despite of this, the updated FMEA approach demonstrated several practical advantages in this analysis, including reduced analysis time, clarity in the categories, and stability with respect to changes in the failure mode list. These aspects are discussed further below.

Figure 2 illustrates the variability in scoring among individual physicists across three FMEA iterations. The initial variability was attributed to differences in the interpretation of failure mode definitions. During the consensus meeting, the PFMs were clarified and rewritten to represent concrete, scenario‐based examples, which facilitated more consistent interpretation and resulted in reduced standard deviations. Notably, even after group discussion, detectability scores continued to exhibit greater inter‐institutional disagreement than the other scoring dimensions.

The persistent variability in scores, even after group discussion, reflects the reality of the varying levels of experience in different institutions that employ different equipment, software and workflows. This observation is consistent with findings reported in the literature.^7^ While this heterogeneity complicates the development of a single consensus score, it also underscores the value of a multi‐institutional approach. The resulting risk analysis captures a broader range of clinical practice patterns compared to any single institutional study.

Given the inability to achieve full consensus, the selection of an appropriate scoring methodology is critical. While averaging scores across participants is common practice in FMEA analysis, this approach may obscure important differences arising from variations in processes and equipment across centers, potentially reducing the relevance of the risk analysis to all participating institutions. After considering average and worst‐case scenarios, we ultimately adopted a severity‐focused approach similar to that described by O'Daniel et al.^26^. This method uses minimum occurrence, maximum severity, and average detectability scores. The rationale for this approach is that high‐severity PFMs warrant attention even when their occurrence is rare, as the consequences of such failures can be catastrophic.

The results in Figure 4 and Table 1 demonstrate that all PFMs selected by the updated FMEA as AP = High and AP = Medium were also selected by the traditional FMEA approach, however the traditional methodology selected an additional 23 PFMs: 7 by the top RPN quartile, 14 by S ≥ 8 and 2 by both the top RPN quartile and the severity criterion. Five PFMs selected by severity alone had very low RPN scores < 27, indicating that despite a high severity score, both occurrence and detectability were scored quite low (e.g., low‐X and high‐X flattening filters installed incorrectly) suggesting that the AP framework may prioritize PFMs in a more actionable fashion within the evaluated dataset.

While the limitations of the RPN have been widely recognized in the FMEA literature outside of medical physics^27^, ^28^, ^29^, ^30^, ^31^ in our study identical RPN values lead to unclear failure mode prioritization. For instance, a PFM such as “Wrong plan version chosen for treatment before pretreatment PSQA: Major dosimetry change.” with the same RPN = 72 as “Collision with Linac components e.g., OBI arms colliding with couch.” but catastrophic severity, arguably warrants same level of attention, yet the RPN formula does not distinguish between these scenarios (see Table 1).

Because of this limitation, the use of an additional method such as a severity threshold, is recommended to prioritize similar RPN results^6^ but the selection of a threshold for action is rather arbitrary^32^ and still requires the analyst to make subjective decisions about prioritization within the selected group (see 9 PFMs with S = 9 outside of the RPN quartile in Table 1). Stamatis^33^ noted that this lack of clear action thresholds represents a fundamental weakness of RPN‐based prioritization across industries.

In our study, the FMEA analysis was repeated across 3 iterations, starting with an initial list of 46 PFMs, subsequently reduced to 37 and later expanded to 63. The iterative exercises showed that by adding or removing PFMs, the RPN quartile boundaries change and alter which failure modes are selected as high priority. This may pose practical challenges for ongoing quality management programs that may need to incorporate new failure modes as processes evolve.

Additionally, determining high‐risk PFMs using the traditional FMEA approach required more than twice the time of the updated approach, as it involves calculating, ranking, and interpreting RPN values, whereas the updated approach simply references the AP table (Figure 1).

A key difference between the AP and RPN risk priority frameworks is that the AP weights occurrence more heavily than detectability introducing a shift in paradigm. Specifically, risk mitigation efforts are directed toward error prevention, as the selection of PFMs are more strongly influenced by occurrence than by detectability. The AP framework's emphasis on prevention over detection aligns with established hierarchies of intervention effectiveness. The ASTRO report “Safety is No Accident”^34^ calls for increased development and adoption of automation in radiation oncology, referencing evidence that forcing functions and computerized systems represent the most effective interventions for improving patient safety^35^ and these interventions translate to lower occurrence scores. Detection‐based QA, while valuable, represents a later and inherently less effective barrier against errors. From the perspective of post‐FMEA interventions, enhancing detection capabilities through additional QA steps may be less effective than reducing the occurrence of PFMs through process redesign. Automation and scripting offer substantial potential for occurrence reduction by standardizing processes and eliminating opportunities for human error.^36^, ^37^ This prevention‐oriented philosophy does not diminish the importance of detection‐based QA, which remains essential for identifying failures that escape upstream control. Rather, it suggests that quality improvement efforts should prioritize occurrence reduction where feasible, reserving detection‐based interventions for PFMs that cannot be effectively prevented.

It is important to recognize that while FMEA considers O, S, and D, none of these factors account for the level of impact that a failure mode may have, specifically referring to the amount of time that an error would go undetected and the number of patients that would be impacted. Some PFMs occur randomly and affect a single patient, while some PFMs occur at a more systemic level, affect a population of patients and have the possibility of remaining undetected until the next cycle of QA. For example, eight out of 35 PFMs in the TPS category and six out of 17 PFMs in the Linac category can affect multiple patients, whereas the data transfer and documentation categories had only PFMs that would impact individual patients. Although not traditionally considered in FMEA analysis, an adaptation of the analysis to medical physics may benefit from adding a fourth factor which reflects the population impact of the failure mode.

Several limitations of our study warrant consideration. First, our multi‐institutional group was drawn from a single province, which aids collaboration but limits generalizability to regions with different clinical practices, equipment configurations, and regulatory requirements. Centers using integrated record‐and‐verify systems, for example, may exhibit different failure mode lists. Second, the representative process map developed by our group may not capture institution‐specific workflow variations that could influence failure mode occurrence and detectability. Third, although consistent with the original aim of this study, i.e., to analyze PFMs detectable through PSQA, the list was not strictly constrained to a single definition of PFM. Instead, it includes errors, contributing causes, and states of equipment degradation, provided they could plausibly be detected through PSQA. While this broader inclusion criterion supports the study objective, it may limit consistency in failure mode definition across the dataset. Finally, the PFM list, initially created through expert brainstorming and later refined with current literature, may introduce selection bias and underrepresentation of unrecognized PFMs. Incorporating incident learning system data may help identify additional modes that might otherwise be overlooked.

The results of this work motivate several avenues for future investigation. Prospective validation studies comparing FMEA predictions with incident learning system data would help establish the predictive accuracy of both the traditional and updated approaches. Application of the updated FMEA to specialized radiotherapy techniques would extend its demonstrated benefits to a broader range of clinical processes. For example, implementation of the same FMEA methodology to PSQA could help determine its effectiveness in identifying various errors leading to improved PSQA efficiency. Development of RT‐specific modifications to the AP lookup table, potentially incorporating population impact as an additional factor, could enhance the relevance of the framework to our field.

## 5 CONCLUSIONS

In this multi‑institutional study, the updated AP‐based FMEA identified all top high‑risk PFMs captured by the traditional RPN‐based FMEA while suggesting a more streamlined, and potentially more consistent prioritization strategy. The traditional FMEA identified additional, lower‑priority modes driven by its quartile‑based and severity‑threshold selection criteria. In contrast, the AP framework emphasizes severity and incorporates well‐defined action levels which may help simplify decision‑making. In this analysis, the AP approach also appeared less sensitive to variability in scoring which may support more consistent prioritization of PFMs particularly in the context of similar multi‐institutional settings where differences in equipment and workflows often challenge consensus‑based approaches. Integrating the AP framework may help streamline clinical FMEA exercises, facilitate risk‑prioritization across institutions, potentially promote more reproducible action plans over time, while helping ensure that the most consequential PFMs receive prompt attention. An updated FMEA approach may also be applied to specialized techniques such as stereotactic radiosurgery, brachytherapy and proton therapy, as well as other RT processes such as chart checking and PSQA, showing its potential to enhance safety and reliability across the broader radiation oncology community.

## AUTHOR CONTRIBUTIONS

Alejandra Rangel made a substantial contribution to the conception of this study and led the drafting, review and integration of the manuscript. Ryan Studinski led and supervised the provincial community of practice group in conducting this work. All authors contributed to the study's conception, design, material preparation, and analysis as well as the writing of the manuscript.

## CONFLICT OF INTEREST STATEMENT

The authors declare no conflicts of interest.

## APPENDIX A

### A.1

Process Map

**Table:**

PFM#	Category	Description	Process Step
1	Planning/TPS	Errors in commissioning/beam model: MLC parameters—Large modelling error (e.g., 3% absolute dosimetric error).	3c
2		Errors in commissioning/beam model: Output factors (e.g., relative field size, reference point dose, 2% dosimetric error).	3c
3		Errors in commissioning/beam model: Wrong Linac model chosen e.g., Clinac instead of TrueBeam (e.g., primary collimator or something else not modeled in TPS).	3c
4		Errors in commissioning/beam model: Bank A is bank B in TPS and reversed in OIS.	3c
5		Errors in CT commissioning: Geometric distances wrong (3 mm).	3c
6		Errors in CT commissioning: CT numbers inaccurate (e.g., HU of water off by 10 HU).	3c
7		Errors in CT commissioning: RED curve programmed incorrectly, gross error resulting in > 2% dose difference.	3c
8		Wrong template used to create plan: Linac characteristics (e.g., SBRT vs. standard Linac).	3a
9		Wrong template used to create plan: Beam parameters (e.g., incorrect energy, FFF vs FF, etc.).	3a
10		Wrong template used to create plan: Target optimization parameters.	3a
11		Wrong template used to create plan: OAR optimization parameters.	3a
12		Wrong settings used in planning system: Calculation grid (e.g., SBRT vs. standard).	3a
13		Wrong settings used in planning system: Dose calculation algorithm (e.g., less rigorous algorithm used).	3a
14		Wrong settings used in planning system: Optimizing too close to surface.	3b
15		Treatment plan errors: Jaw incorrectly blocking PTV.	3b
16		Treatment plan errors: Jaw incorrectly exposing OAR.	3b
17		Treatment plan errors: Target contours have stray voxels.	2b
18		Treatment plan errors: Jaw tracking was forgotten.	3b
19		Treatment plan too complex: High MU factor (MU/Gy above typical).	3b
20		Treatment plan too complex: Small leaf gap.	3b
21		Treatment plan too complex: Fields (defined by jaws) smaller than modeled.	3c
22		Treatment plan not calculated on the correct version of CT scan: Older scan after weight loss.	3a
23		Treatment plan not calculated on the correct version of CT scan: Wrong breathing phase.	3a
24		Treatment plan not calculated on the correct version of CT scan: Wrong bladder filling.	3a
25		Treatment plan not calculated with a proper RED curve.	3c
26		Treatment plan not calculated with proper density override/couch.	3c
27		Configuration difference between plan and Linac: Beam parameters (PDD, profiles) above guidelines.	3c
28		Configuration difference between plan and Linac: Linac configuration differences (e.g., gantry or collimator angle).	3c
29		Configuration difference between plan and Linac: Couch parameters (e.g., tabletop movement, couch kick).	3c
30		Configuration difference between plan and Linac: Planned on wrong Linac (e.g., the Linac does not have the planned dynamic wedge, or it has 10 MV while the plan uses 18 MV).	3a
31		Configuration difference between plan and Linac: SBRT case planned on non‐SBRT Linac where QA is more relaxed.	3a
32		Configuration difference between plan and Linac: Case intended for an HD‐MLC was planned on STD‐MLC Linac.	3a
33		Configuration difference between plan and Linac: Plan has more MU than the Linac is configured to deliver but is planned on the right Linac.	3b
34		Gross failure of planning system: Failing to recalculate MU after a parameter has been changed in software (e.g., 60 Gy/30 changed to 60 Gy/8 but no MU update).	3c
35		Gross failure of planning system: Failing to update dose distribution after parameter change (e.g., energy changed from 6 to 18MV).	3c
36	Delivery/linac	Major degradation of linear accelerator: Ion chamber leaking.	5d
37		Major degradation of linear accelerator: Target melting.	5d
38		Major degradation of linear accelerator: Vacuum degradation.	5d
39		Linac performance above action level: Output at around action level (5%)	5d
40		Linac performance above action level: Flatness/symmetry at around action level (5%).	5d
41		Linac performance above action level: MLC calibration offset of ∼3 mm.	5d
42		Linac performance above action level: MLC calibration gain of ∼1 mm/5 cm.	5d
43		Linac performance above action level: Loss of leaf speed 2 cm/sec.	5d
44		Linac can't deliver the plan/ Linac interlock (e.g., machine related, MLC speed or dose rate, unable to clear).	5d
45		Linac can't deliver the plan/ Linac interlock can be cleared.	5d
46		Collision: Touch guard goes off.	5a/5d
47		Collision: Linac components (e.g., OBI arms colliding with couch).	5a/5d
48		Collision: Patient.	5a/5d
49		Major Linac failure (e.g., wrong flattening filter moves into place).	5d
50		Wrong physical component installed (x‐low and x‐high flattening filters reversed or Linac Pb shielding left out).	5d
51		Incorrect or limited out‐of‐field dose (e.g., 2 cm beyond field edge) > 5% absolute dose difference.	5d
52		Linac performance is not the same as commissioning: Other VMAT parameters like gantry speed or dose rate.	5d
53	Documentation and data transfer	Wrong plan version chosen for treatment before pretreatment PSQA: Major dosimetry change (e.g., wrong prescription relative to intended, a change of > 100 cGy/day).	4b
54		Wrong plan version chosen for treatment before pretreatment PSQA: Moderate dosimetry change (e.g., plan was updated, plan with 10% reduction to specific OAR dose not used).	4b
55		Wrong plan version chosen for treatment before pretreatment PSQA: Minor dosimetry change (e.g., 7% hotspots reduced to 3% however “hot” plan was treat‐approved instead of “cold” plan).	4b
56		Wrong plan version chosen for treatment after pretreatment PSQA: Major dosimetry change (e.g., wrong prescription relative to intended, a change of > 100 cGy/day, no follow‐up PSQA after treatment starts).	5b
57		Wrong plan version chosen for treatment after pretreatment PSQA: Moderate dosimetry change (e.g., plan was updated, plan with 10% reduction to specific OAR dose not used, no follow up PSQA after treatment starts).	5b
58		Wrong plan version chosen for treatment after pretreatment PSQA: Minor dosimetry change (e.g., 7% hotspots reduced to 3% however “hot” plan was treat‐approved instead of “cold” plan, no follow‐up PSQA).	5b
59		Plan modified after treatment has begun (including first‐fraction treatment checks): Moderate dosimetry change (no follow up PSQA after patient starts).	5b
60		Plan modified after treatment has begun: Minor dosimetry change (no follow‐up PSQA after treatment starts).	5b
61		Minor data transfer error while importing into OIS (e.g., MLC positions or MUs rounded off).	4b
62		Major data transfer error while importing into OIS (e.g., beam not transferred to the OIS).	4b
63		Data transfer error from OIS to Linac (e.g., OIS values exceed what Linac can do).	5c

* PFMs unlikely to be detected by PSQA were intentionally excluded from the list.
